# Supplementary figures and images for: MDM2 inhibition in combination with endocrine therapy and CDK4/6 inhibition for the treatment of ER-positive breast cancer
Source: Breast Cancer Res. 2020 Aug 12;22:87. doi: 10.1186/s13058-020-01318-2 (PMC7425060; doi:10.1186/s13058-020-01318-2)

Figure S1

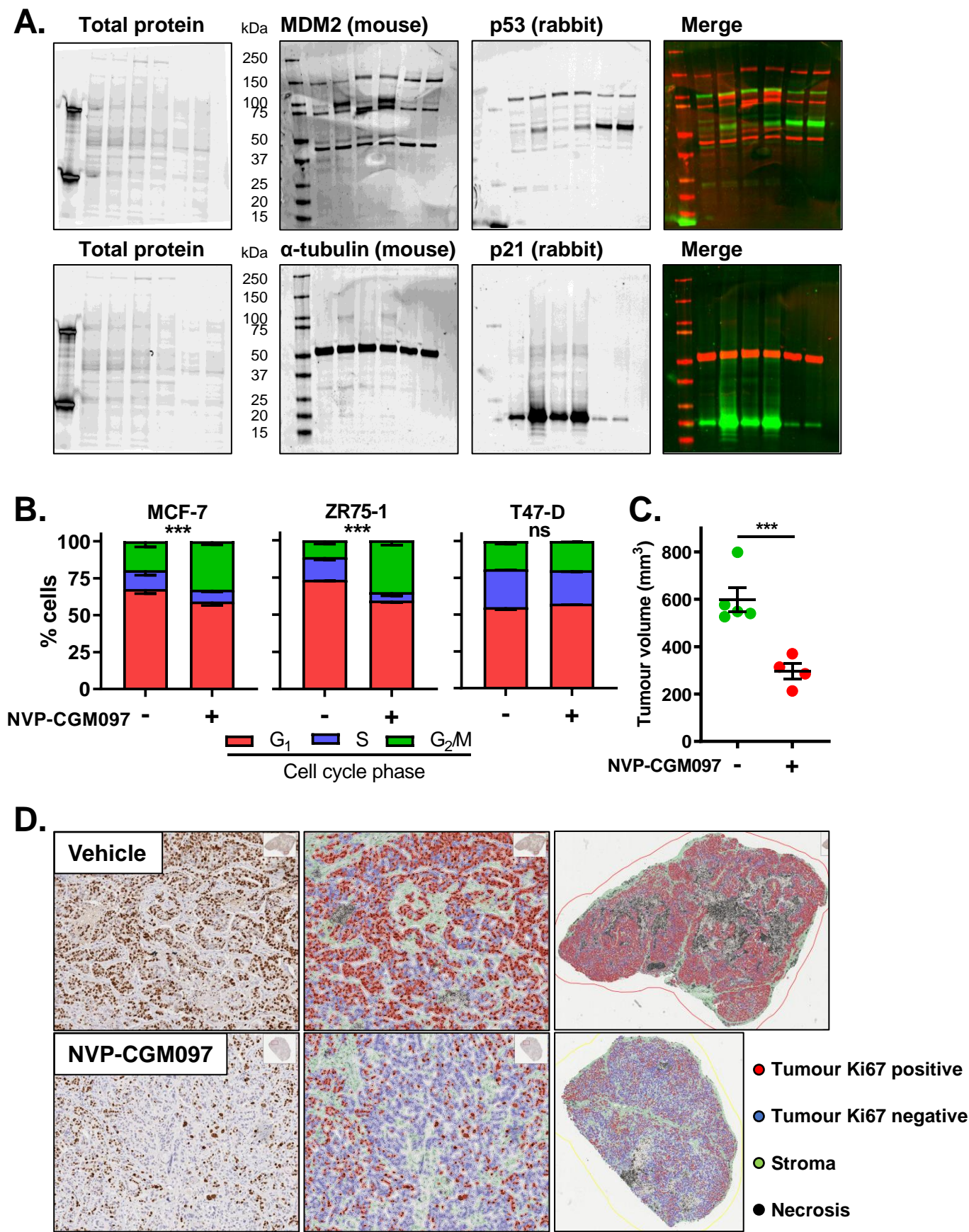

Figure S2

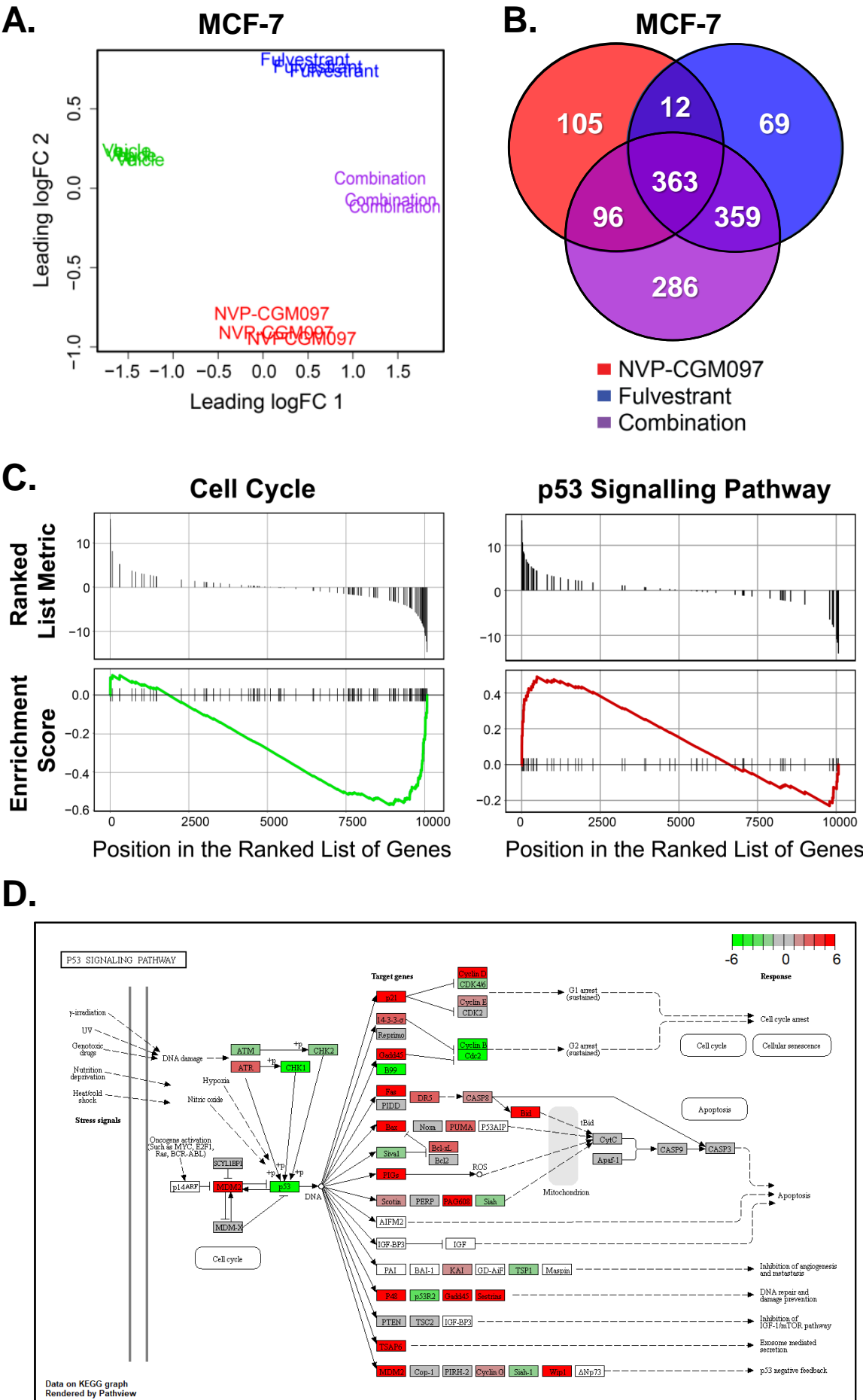

**A.**

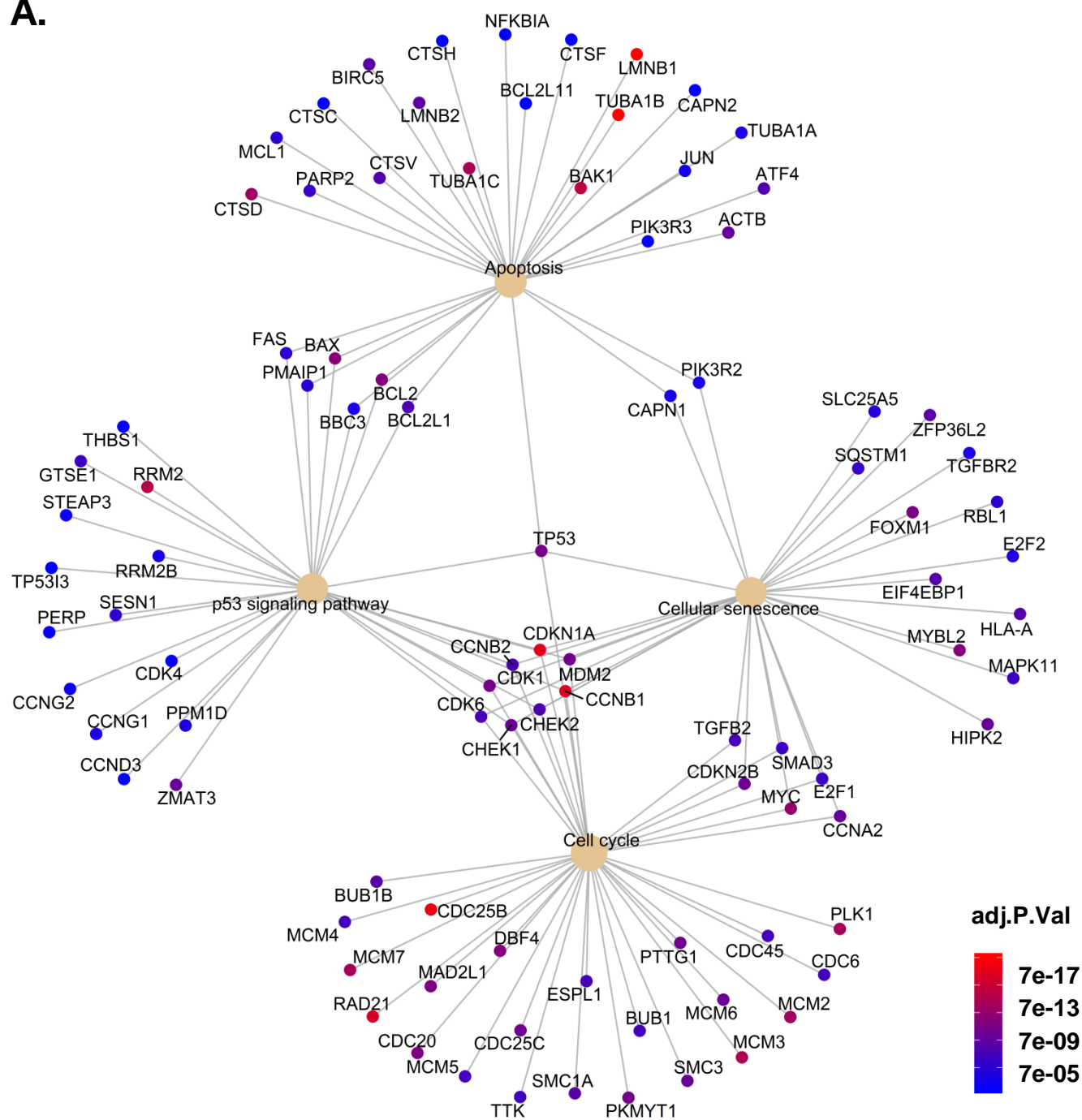

Figure S4

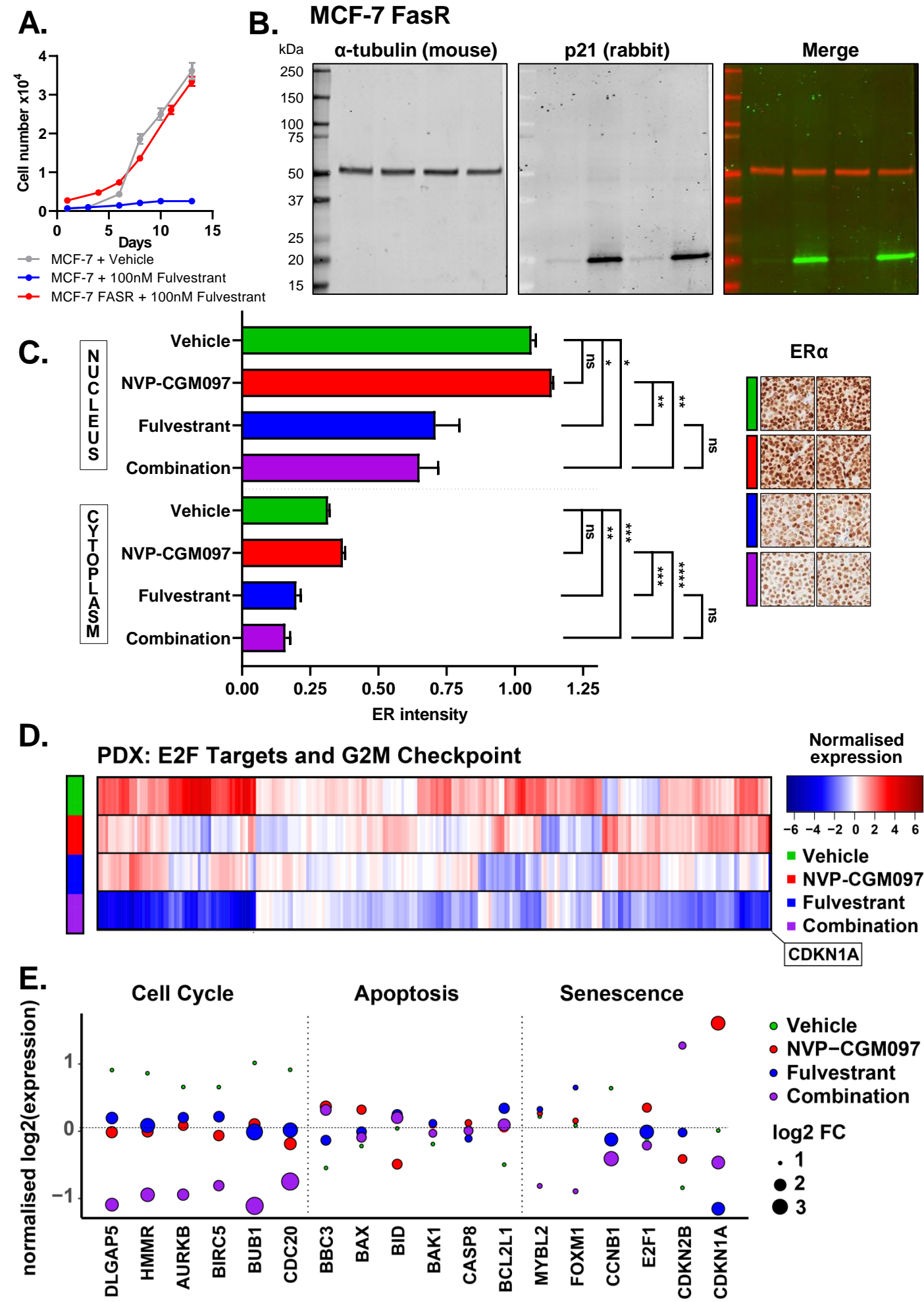

Figure S5

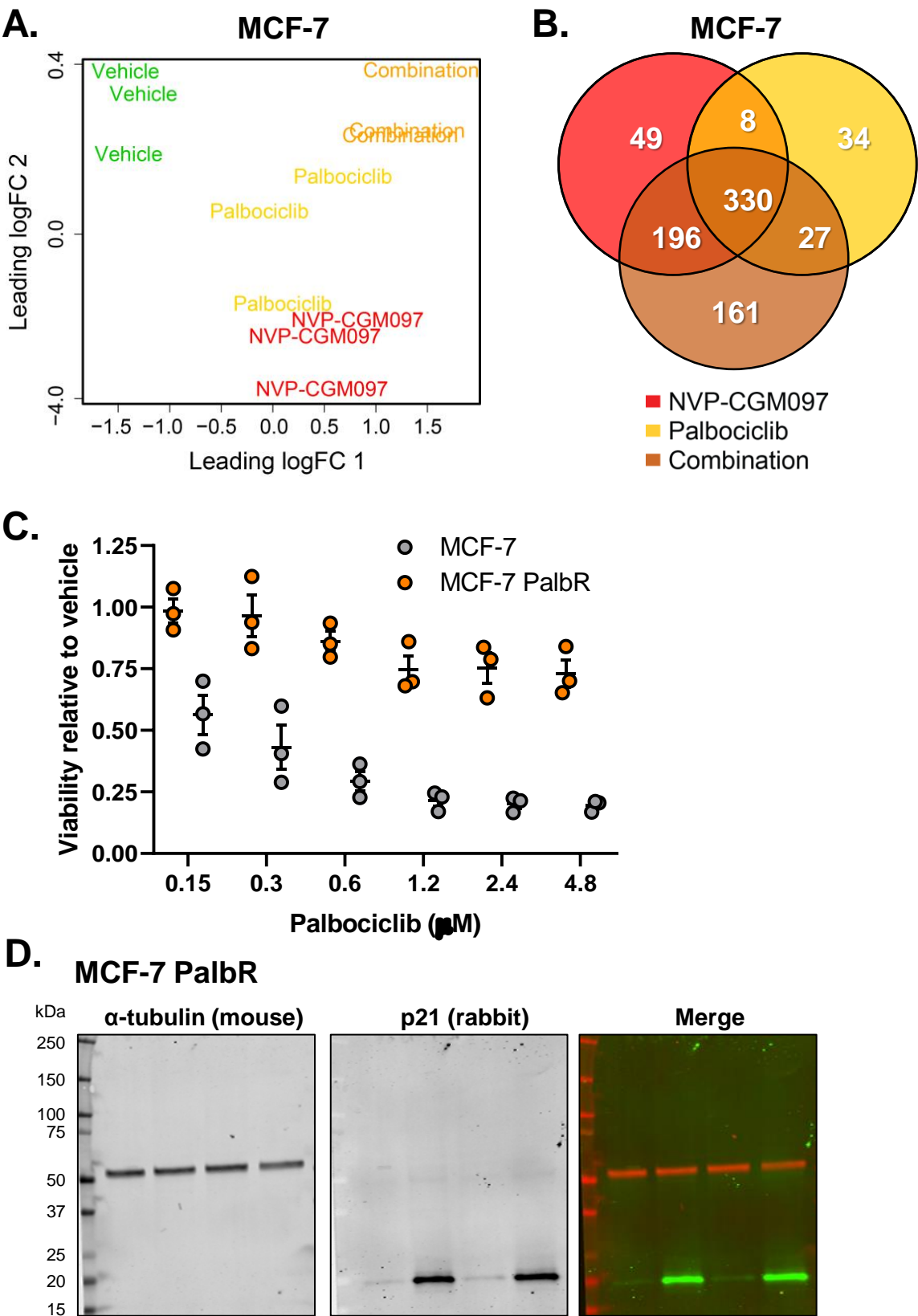

Supplement: Supplementary file 2 — Additional file 2: Fig. S1 MDM2 inhibition activates p53 and reduces tumour proliferation in vitro and in vivo. A. Full gel and blot scans for the Western blots shown in Fig. 1b. Total protein was visualised using BioRad stain-free imaging technology according to the manufacturer’s instructions. B. Analysis of cell cycle phase using flow cytometry to quantify propidium iodide staining of genomic DNA shows significant alterations to cell cycle phase distribution in p53wt models consistent with arrest in both G1 and G2 after incubation for 48 hours with 1μM NVP-CGM097. Red = G1 (bottom), blue = S (middle), green = G2/M (top). Statistical significance from χ2 test using the vehicle treated profile as the expected value is indicated. C. NVP-CGM097 (50mg/kg daily, red) significantly inhibited tumour volumes compared to vehicle (2% DMSO daily, green) at endpoint. Final tumour volumes were compared using two-tailed T test to determine significance. D. Representative images of Ki-67 quantification of endpoint tumours in Qupath software showing the classification of different tissue compartments: tumour (red and blue), stroma (green), and necrosis (black); and detection of Ki-67 negative and positive tumour cells. A single classifier was applied to all tumour sections. Fig. S2. NVP-CGM097 treatment causes gene expression changes in cell cycle and p53 pathways in vitro. A. Multidimensional scaling (MDS) plot showing the level of sample similarity between MCF-7 cell lines treated with vehicle, NVP-CGM097, fulvestrant and combination therapy (NVP-CGM097 plus fulvestrant). B. Venn diagram showing the overlap between differentially expressed genes (adjusted p-value < 0.05- and 2-fold change) induced by treatment in MCF-7 cell lines. C. KEGG pathway analysis using RNA-Seq transcriptomic data shows a significant negative enrichment of Cell Cycle regulation in MCF-7 cell lines following 48 hours of treatment with 1μM NVP-CGM097 and positive enrichment of p53 Signalling Pathway. D. K [file 13058_2020_1318_MOESM2_ESM.pdf]
